# Role of Selective Sodium-Glucose Co-Transporter-2 Inhibitors in Managing Cardio-Renal Complications in Type 2 Diabetes Mellitus: Beyond Glycemic Control

**DOI:** 10.7759/cureus.17452

**Published:** 2021-08-26

**Authors:** Chike B Onyali, Comfort Anim-Koranteng, Hira E Shah, Nitin Bhawnani, Aarthi Ethirajulu, Almothana Alkasabera, Jihan A Mostafa

**Affiliations:** 1 Internal Medicine, California Institute of Behavioral Neurosciences & Psychology, Fairfield, USA; 2 Medicine, California Institute of Behavioral Neurosciences & Psychology, Fairfield, USA; 3 General Medicine, California Institute of Behavioral Neurosciences & Psychology, Fairfield, USA; 4 Psychiatry/Cognitive Behavioural Psychotherapy, California Institute of Behavioral Neurosciences & Psychology, Fairfield, USA

**Keywords:** sglt2, diabetes mellitus type 2, chronic kidney disease (ckd), sodium-glucose co-transporter 2 inhibitor, cardio vascular disease, human pathophysiology, pharmacology

## Abstract

Chronic kidney disease (CKD) and cardiovascular complications are the leading causes of death in type 2 diabetes mellitus. Apart from the standard therapy, which includes angiotensin-converting enzyme inhibitors (ACEi), angiotensin receptor blockers (ARBs), lipid-lowering medication, and anti-platelet therapy, the new group of drugs termed the 'sodium-glucose co-transporter-2 (SGLT2) inhibitors' have shown promising results in managing complications arising from the cardiovascular and renal systems in diabetics. This article attempts to highlight the role and mechanism of action of this class of drugs.

We reviewed 127 articles and analyses of randomized controlled trials using several drugs in the SGLT2 inhibitor family (sotagliflozin, canagliflozin, dapagliflozin, tofogliflozin) over the past five years, out of which 58 met the criteria and aim of the study. These articles were retrieved from PubMed, Google Scholar, and Medline data sources and assessed for quality using the assessment of multiple systematic reviews (AMSTAR) checklist and Cochrane risk-of-bias tool. Results from the review showed significant benefits in reducing progressive renal decline, blood pressure control, heart failure hospitalization, death from renal or cardiovascular complications, myocardial infarction, and stroke. This benefit is also seen in non-diabetic patients, hence postulating that these effects may not be solely due to glycemic control. There are several mechanisms with which it achieves this benefit with the most significant being its role on intraglomerular pressure. Other pathways include blood pressure control, natriuresis, ventricular remodeling, erythropoiesis, lipid metabolism, plasma volume, and electrolyte imbalance.

It is clear that the role of SGLT2 inhibitors isn’t limited to glycemic control and they can achieve a wide array of functions by affecting different systems. More studies need to be done to completely understand this medication to improve the quality of life in diabetic and non-diabetic patients living with CKD and cardiovascular complications. The pharmacokinetics of this drug could also help set the basis for newer medications.

## Introduction and background

Diabetes is a life-long disease that affects millions of people from across the globe. Roughly 422 million people were living with diabetes in 2014, which is almost a three-fold rise from figures in 1980 [1], and by 2030, this figure is expected to increase to over 430 million [2]. A 5% rise in premature mortality was seen from 2000 to 2016, despite rising knowledge and interventions. It’s possible that increased awareness and diagnostic qualities also played a role in this rise. The complications related to this condition affect mostly the cardiovascular and renal systems and are responsible for about 40% of the total number of patients with chronic kidney disease (CKD) worldwide [3]. This effect increases the risk of cardiovascular disease (CVD), end-stage renal disease, and mortality. Thus, the utmost priority should be given to understanding and properly managing these complications. 

Despite the use of renin-angiotensin inhibitors (e.g. angiotensin-converting enzyme inhibitors (ACEi) and angiotensin receptor blockers (ARB)), lipid-lowering and anti-platelet drugs, which are the standard therapy in managing these diseases, mortality remains high and it is clear that intensive research needs to be done to meet up with the demands of handling these obstacles. Sodium-glucose co-transporter-2 (SGLT2) inhibitors are the latest group of medications approved for managing diabetes, following documented benefits in several trials. Recent studies have shown significant effects in achieving glycemic control, reducing renal and cardiovascular complications. In some common cardiovascular outcome trials (CVOTs) such as the dapagliflozin effect on cardiovascular events led by the 'Thrombolysis in Myocardial Infarction' study group (DECLARE-TIMI 58), empagliflozin cardiovascular outcome event trial in Type 2 diabetes mellitus patients removing excess glucose (EMPA-REG), and the canagliflozin cardiovascular assessment study (CANVAS), SGLT2 inhibitors showed protective cardio-renal capabilities in diabetic patients with heart failure, CKD, and cardiovascular risk factors [4-7]. More studies are currently exploring its effect on heart failure (reduced ejection and normal ejection fraction) and CKD [8]. 

The mechanisms by which these effects on the cardio-renal system are achieved are not quite understood. In the renal system, some theories postulate the benefits are directly linked to its role in reducing intraglomerular pressure. While in the cardiac system, it is thought to be due to its action on cardiac progenitor cells, arterial stiffness, hematocrit levels, fluid, and electrolyte balance. 

 A comprehensive review of the effect and actions of the SGLT2 inhibitors on the cardio-renal system is important to effectively manage these complications and reduce mortality. In this paper, we aim to analyze the effects of this class of drugs on these systems as well as to provide a thorough review of its mechanism of action. 

 A better understanding of this process will give us more insight into achieving maximum success with this class of drugs and also set a benchmark for newer medications. 

## Review

Effect of SGLT2 inhibitors on the renal system

Diabetes causes a significant percentage of CKD, a condition that has affected millions of people worldwide [1]. Managing this complication can be difficult and one of the limitations in patients with CKD is that it is usually asymptomatic until a significant amount of renal function has been lost, which subsequently leads to a steady reduction in renal function till end-stage renal disease (ESRD), with renal transplant being the sole treatment modality in this late phase. However, renal transplant is very expensive and access to donors and quality of life is a major issue in some patients, hence early detection and prevention of decline is important. Estimated glomerular filtration rate (GFR) is used to assess and monitor renal function in these patients. 

Blood pressure control is known to significantly reduce renal decline and, for a while, the ARBs and the ACEi were the only class of drugs that slowed down renal decline in diabetics with CKD. This study aims to highlight the significant effects of SGLT-2 inhibitors and attempts to understand their mechanism of action. 

Effects on Intraglomerular Pressure

In the canagliflozin and renal events in diabetes with established nephropathy clinical evaluation (CREDENCE) trial, i.e., estimated GFR of 30 to <90ml/min/1.73m2 or albuminuria clinical evaluation, that was aborted early after the primary endpoints were reached, the SGLT2 inhibitors were effective in delaying the progression of CKD and reducing albuminuria. There was a 30% reduction in the risk of ESRD, doubling of creatinine, and death from renal or cardiovascular pathways [9,10]. Several theories have stated how it achieves this response, including effects on intraglomerular pressure, glomerular size selectivity, tubular inflammation, and electrolyte management. [11] The meta-analysis from the EMPA-REG, CANVAS, and DECLARE-TIMI 58 studies showed that the absolute risk of progressive renal decline, ESRD, or death from renal causes dropped by 45% with SGLT 2 inhibitors vs placebo, and this role was extended to those with established CVD or multiple cardiovascular risk factors. [12-15] 

**Figure 1:**
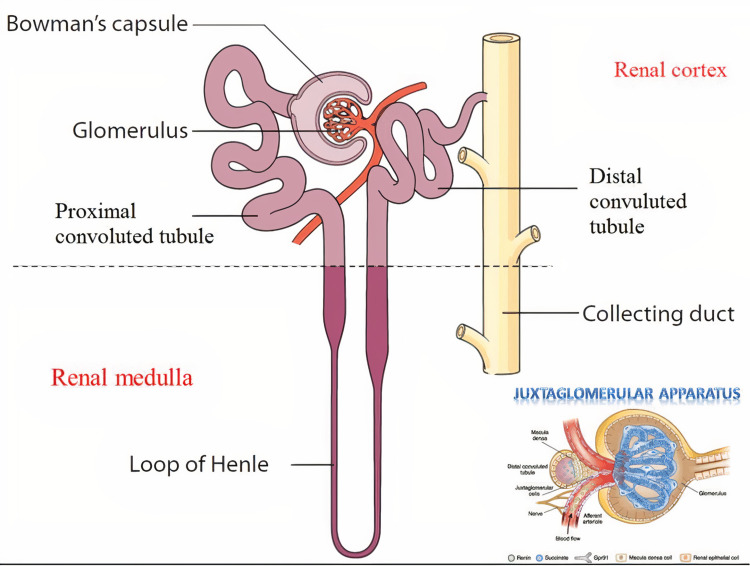
Segment of a nephron and its juxtaglomerular apparatus Source: El-Reshaid K et al. [19]. Permission taken.

The juxtaglomerular apparatus (JGA) is a segment of the nephron that regulates systemic blood pressure and comprises the juxtaglomerular cells, macula densa (modified distal convoluted tubule cells), and lacis cells [16]. SGLT-2 inhibitors work by inhibiting the co-transport of glucose with sodium at the proximal tubule of the kidney, leading to a rise in the concentration of sodium being delivered to the macula densa. The macula densa then causes afferent arteriolar vasoconstriction and sends feedback to the JGA. These responses reduce renal blood flow and intraglomerular pressure, causing an acute decrease in GFR with eventual stabilization of GFR, which translates to improved proteinuria, and renal function [17]. Meta-analysis of clinical trials also showed that a 30% decrease in proteinuria translates to about a 30% decrease in the risk of ESRD [18]. Figure 1 shows the segment of the nephron and its JGA.

**Figure 2:**
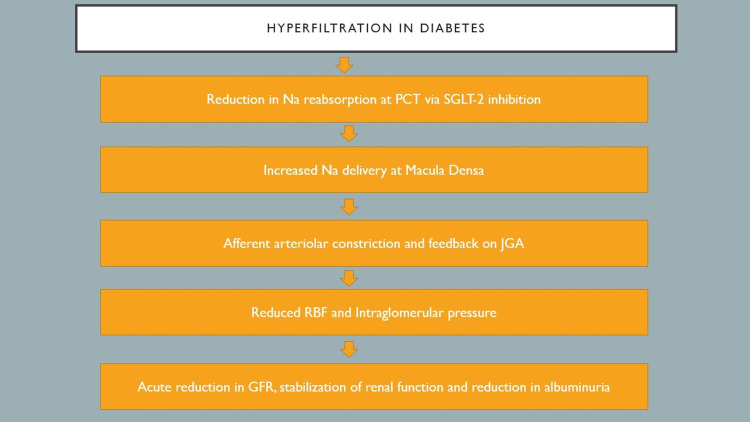
Showing the response of the nephron to SGLT-2 inhibition PCT: Proximal convoluted tubule, SGLT-2: Sodium-glucose co-transporter-2, Na: Sodium, JGA: Juxtaglomerular apparatus, RBF: Renal blood flow

Figure 2 describes the effects of SGLT-2 inhibitors on the nephron

Effects on Homeostasis 

Its role on plasma volume is primarily via osmotic diuresis, which leads to blood pressure reduction, limiting further renal and cardiovascular damage. It has been postulated to also affect serum osmolality, particularly via sodium metabolism with resultant effects on the renin-angiotensin mechanism. Some studies showed that using empagliflozin 25mg was associated with increased urinary glucose excretion, transient natriuresis, and urine volume with no significant change in renin-angiotensin system [20]. In patients with CKD, with or without diabetes, the risk of sustained decrease in estimated GFR by at least 50%, ESRD, or death from renal causes was significantly lower with dapagliflozin compared to placebo [21]. 

Worthy of note is that single and multiple administrations of tofogliflozin achieved the same effect across various ranges of renal impairment, so dose titrations are not necessary [22,23], hence limiting possible side effects associated with higher doses. The absolute benefits of this drug were seen in the group with lower estimated GFR (30 to <45ml/min per 1.73m2). In the CANVAS Program, the relative benefits were consistent over varying levels of proteinuria while the absolute benefits were proportionate to increasing levels of proteinuria. This translates into improved outcomes for individuals with severely impaired renal function, hence, delaying the progression of CKD and decreasing the chances of tilting to ESRD, which is the goal of therapy. [24] 

Effect on Erythropoiesis 

One of the major problems associated with CKD is anemia. The presence of anemia complicates the clinical scenario and worsens the prognosis. An analysis of the CREDENCE trial by Oshima et.al. noted that the results of 'anemia outcome or the initiation of anemic treatment (iron preparation, erythropoietic stimulating agents)' was lower in the canagliflozin arm than the placebo group. This reduces the need for frequent blood transfusions and the complications associated with this procedure, further reducing hospital visits, emergencies, and improving survival outcomes in these patients. [25] 

From the above studies, the SGLT2 inhibitors show increasing efficacy in patients with declining renal function. This appears to be independent of glycemic control, hence, its possible use in all patients with CKD. The lower limit of estimated GFR used in most studies was between 25-30ml/min/1.73m2 and more trials are being done to establish their role in lower estimated GFR levels and ESRD. There are lots of possible pathways that explain its mechanism of action but what is prominent across most studies are their effects on intra-glomerular pressure and effective glycemic control with a very low risk of hypoglycemia. Worthy of note with this class of drugs is their role in improving erythropoiesis, which translates to better outcomes and mortality in these patients. Some studies show a single dose recommendation with tofogliflozin achieves a similar effect [21,22], However, incremental dosing techniques are used with other medications in this group to achieve better control, as noted with empagliflozin. 

In the CREDENCE trial by Cannot et al., the study involved canagliflozin in a double-blinded, randomized controlled trial using a total no. Of 4401 patients, 99.1% completed the trial, quality assessment using the Cochrane risk-of-bias tool was 93.7% in comparison to a double-blinded randomized control trial with dapagliflozin by Heerspink et al. involving 4304 patients of which 99.7% completed the study, quality assessment using CRBT was 87.5%. Although both studies had primary outcomes of ESRD and death from renal or cardiovascular causes, Heerspink et al. was also noted to have included a sustained decline in estimated GFR of at least 50% as part of its primary outcome. In post hoc analysis of the CREDENCE trial by Jardine et al., quality assessment using AMSTAR checklist was 78.5% while the quality assessment of the CANVAS trial by Neuen et al. using the same AMSTAR checklist was 82.1%. 

Effects of SGLT-2 inhibitors on the cardiovascular system

Cardiovascular complications are the leading cause of death from diabetes. They further worsen other pre-existing and renal co-morbidities that are usually present in this group of patients. There is a wide range of cardiovascular complications associated with diabetes that include but are not limited to death, stroke, coronary artery disease, myocardial infarction, and heart failure. The burden of these complications on the patient and society is so immense that adequate interventions have to be formulated to deal with these problems. Several drugs are being used to help curtail these health conditions such as lipid-lowering drugs, antiplatelet therapy, and most recently the SGLT 2 inhibitors, which were originally used for glycemic control in diabetic patients but have shown significant effects in managing some of these complications, directly and indirectly. These benefits have also been noted to extend to the non-diabetic population. 

Effect on Blood pressure and Heart failure 

The inhibition of the sodium-glucose co-transporter in the proximal tubule promotes osmotic diuresis which decreases plasma volume, thereby reducing preload and ventricular strain. Since these parameters are significant factors in the pathophysiology of heart failure, it is a possible explanation for the benefits seen in these patients. SGLT2 inhibitors are also known to decrease systolic blood pressure through the same mechanism, further limiting the stress on the myocardium. 

**Figure 3:**
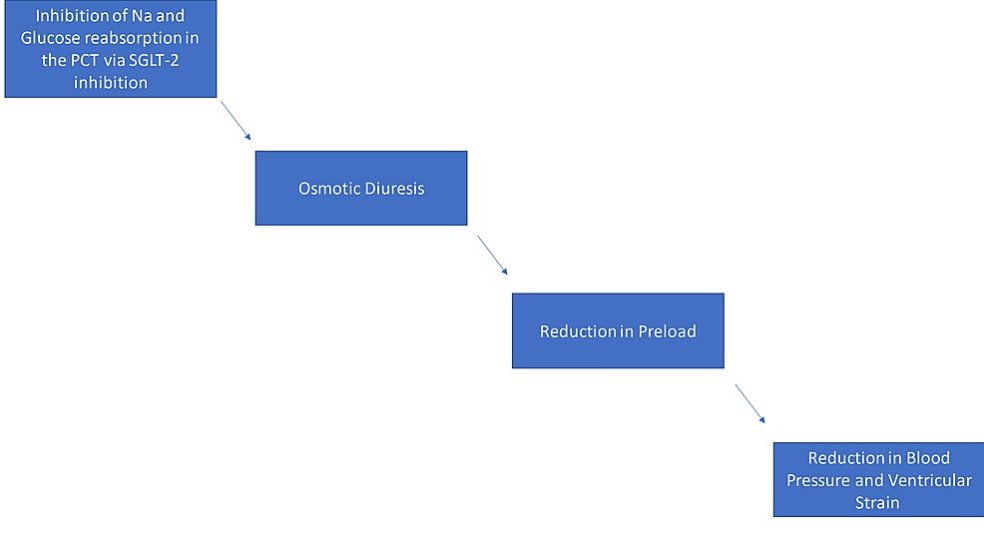
Role of SGLT2 inhibitors on blood pressure PCT: Proximal convoluted tubule, Na: Sodium, SGLT2: sodium-glucose co-transporter-2

 Similarly, in the CVOTs using empagliflozin (EMPA-REG) and canagliflozin (CANVAS), the risk of a three-point major adverse cardiovascular event (MACE); which includes cardiovascular death, non-fatal stroke, non-fatal myocardial infarction, was significantly reduced by 14% in the drug arm versus the placebo. Both studies involved Type 2 diabetes mellitus patients who had established CVD, while the CANVAS study involved patients with CVD risk factors. The DECLARE-TIMI 58 trial using dapagliflozin also showed a reduction in the 3-point MACE with greater effects in the population with previous myocardial infarction [12-15]. Meta-analysis of these CVOTs showed a decrease in the risk of 3-point MACE by 11% compared to placebo in patients with established atherosclerotic CVD but there was no extended benefit to those with multiple risk factors. However, there was a major decline in cardiovascular death and heart failure hospitalization [9,10]. Figure 3 summarizes the effect of SGLT2 on blood pressure and ventricular strain.

Effect on Myocardium 

 In the post hoc analysis of the EMPA-REG trial by Inzucchi et al., the reduction in cardiovascular deaths in patients receiving SGLT2 inhibitors occurred early, and as such it is unlikely that the primary mechanism by which it achieved this short-term advantage is via its role on atherogenesis [26]. A better explanation of this effect is likely due to its action on plasma volume. However, the benefits on cardiovascular death were sustained throughout the trial, so it is also impossible to rule out potential actions on other long-term factors e.g atherogenesis, myocardium, and ventricular remodeling, which have been known to play significant roles in cardiovascular mortality. In the same analysis, the most important mediators in reducing death from cardiovascular causes were changes in markers of plasma volume, with an increase in hematocrit (hemoglobin) levels playing the most important role [26]. Further analysis also showed that despite using different doses of empagliflozin (10mg and 25mg), there were equal cardiovascular benefits across both dosage groups. 

In the effects of empagliflozin on cardiac structure in patients with type 2 diabetes (EMPA-HEART) study, which was a randomized controlled trial using empagliflozin involving patients with type 2 diabetes mellitus and stable coronary artery disease, with a significant number with heart failure, there was a reduction in left ventricular mass index over a six-month period [27-30]. What is also important to note in this trial is that the action on ventricular mass was not related to preload, blood pressure, or autonomic changes. This highlights an independent action on the myocardium, which may be of importance in managing other heart conditions including ischemic heart disease and cardiomyopathies [27-30]. 

Effect on Atherogenesis 

In an attempt to fully understand the role of this drug on the atherosclerotic pathway, Katami et al. attempted to study the effect of tofogliflozin on the progression of carotid atherosclerosis. In the prospective randomized study, Type 2 diabetic patients with no apparent CVD history were placed on either tofogliflozin or placebo, carotid atherosclerosis was assessed using carotid intima-media thickness (IMT). Results showed an increase in (high-density lipoprotein) HDL-c with no apparent change in carotid IMT between the two groups [31]. Apart from a lack of evidence showing the connection between carotid IMT and atherosclerosis, a recent meta-analysis showed that there is no correlation between both factors. [31] 

In a double-blinded randomized controlled study conducted in 2019, patients with type 2 diabetes mellitus and a history of coronary artery disease who received empagliflozin showed a reduction in cardiovascular death by 48%, all-cause mortality by 43%, and heart failure hospitalization by 50% [32]. In patients already taking ertugliflozin compared to placebo, death from cardiovascular causes or hospitalization for heart failure occurred in 444 of 5499 (8.1%) and 250 of 2747 (9.1%) [33].

Concerns have been raised about the effect of these drugs when used in combination with other anti-glycemic medications, particularly as it affects glomerular filtration. However, Ikonomidis et al. showed that there was a synergistic effect in patients receiving other drugs in combination with SGLT2 inhibitors [34], these medications include insulin, glucagon-like peptide-1 (GLP-1) agonists, and metformin. The benefits of these drugs have also been shown to be effective across different races. There was a prominent reduction in the risk of cardiovascular adverse events occurring amongst Asians, Middle Easterners, and North Americans. [34] 

From the review of the studies, it is clear that SGLT2 inhibitors have a pronounced effect on the cardiovascular system, particularly in those with already established CVD. It has been shown to reduce the risk of cardiovascular death, myocardial infarction, stroke, heart failure hospitalization, and blood pressure management. It achieves this possibly due to its effect on plasma volume, via natriuresis, leading to a reduction in the preload, which causes lowering of the blood pressure, but there are not enough studies on its role in serum osmolality and electrolyte balance. Other studies have highlighted a possible role in myocardial remodeling and erythropoiesis, which calls for its use in heart failure and acute coronary syndromes, although in this regard, more research needs to be done. Another possible route of action on the cardiovascular system is through an increase in HDL levels, which is associated with good cardiovascular outcomes. A synergistic effect has also been noted when this class of drugs is given in combination with other therapies. In the effect of sotagliflozin on cardiovascular and renal events in patients with type 2 diabetes and moderate renal impairment who are at cardiovascular risk (SCORED) trial by Bhatt et al. Accessing the efficacy of sotagliflozin in type 2 diabetes mellitus with CKD. It involved 10,584 patients (5292 in the sotagliflozin arm). The primary end-point was deaths from cardiovascular causes, hospitalization for heart failure, and urgent visits for heart failure. The estimated GFR of the participants was between 25-60 ml/min/1.73m2. Meanwhile, the CREDENCE trial using canagliflozin, involved 4401 patients with type 2 diabetes mellitus and CKD with cardiovascular primary end-point of cardiovascular death, non-fatal myocardial infarction or stroke, and hospitalization for heart failure, while the estimated GFR of the study group ranged between 30 to < 90ml/min/1.73m2. The post hoc analysis by Davies et al. involving canagliflozin used 2313 patients with varying cardiovascular risk factors while the EMPA-REG trial involved 7020 study groups with varying cardiovascular risk factors [35].

Limitations

Inclusion criteria included randomized controlled trials, review articles, and publications done in the past five years. Only three databases (PubMed, Medline, and Google Scholar) were used while reviewing articles. This article did not comprehensively review studies done in certain populations like Africans, Asians, and Middle Easterners.

## Conclusions

SGLT2 inhibitors have shown to be effective in reducing renal and cardiovascular complications. This article aims to understand these benefits and their mechanism of action. It is been noted that renal decline and cardiovascular complications are significantly reduced in diabetes as well as non-diabetics. Besides glycemic control, the effect on intraglomerular pressure and plasma volume are more obvious pathways by which these drugs work in both groups. Other more probable pathways include the effects on erythropoiesis, natriuresis, ventricular re-modeling, electrolyte, and lipid balance. 

A better understanding of the action of this class of drugs will help to direct the proper use to achieve maximum potential in high-risk patients, while also reducing mortality, morbidity, and side effects. The benefits of these drugs are independent of glycemic control as seen in CKD patients without diabetes as well as its effects on the myocardium, therefore, more randomized clinical trials need to be done in the non-diabetic population. This could be potentially groundbreaking and perhaps offer more explanation to its pharmacokinetics. 
